# Effects of *Varroa destructor* on *Apis mellifera capensis* Drone Sperm Quality Characteristics and Colony Demographics

**DOI:** 10.1155/japr/8285152

**Published:** 2026-09-01

**Authors:** Retha Christina Magrietha Kotze, Janice Faith Murray, Gerhard van der Horst, Mike Allsopp

**Affiliations:** ^1^ Comparative Spermatology Laboratory Group, Department of Medical Bioscience, University of the Western Cape, Bellville, South Africa, uwc.ac.za; ^2^ Department of Dietetics and Nutrition, University of the Western Cape, Bellville, South Africa, uwc.ac.za; ^3^ Agricultural Research Council, Plant Protection Research Institute, Pretoria, South Africa, entecra.it

**Keywords:** *Apis mellifera capensis*, drone, infestation, sperm functionality, sperm motility, *Varroa destructor*

## Abstract

*Varroa destructor (V. destructor)* threatens honeybee colonies and negatively affects drone weight and survival. Therefore, this mite species poses a risk to the reproductive potential of drones, and subsequently colony health and performance. However, studies on the impact of *V. destructor* infestation on sperm characteristics are limited, and results appear to be inconclusive. This study aimed to investigate the effect of *V. destructor* infestation on multiple sperm quality characteristics that are regarded as important for successful fertilization. In this cross‐sectional study, conducted in the Western Cape, South Africa, during the summer of 2021, 53 sexually mature *Apis mellifera capensis (A.m. capensis)* were harvested. Analysis of semen included functional (total motility, progressive motility and swimming speeds; kinematic parameters) and structural sperm parameters (sperm head, tail, total length) using a computer‐aided sperm analysis system. Drone weight and demographic information of colonies were also assessed. Three infestation groups were compared, i.e., < 1% (*n* = 4 colonies) (median: 0.33%; range: 0.00–0.40%), 1%–2% (*n* = 2 colonies) (median: 1.07%; range: 1.00%–1.45%) and 10% (*n* = 2 colonies) (10.0% ± 0.00%). In this study, *V. destructor* infestation had minor, non‐significant effects on drone weight (11% lower in highly infested drones; *p* = 0.123) and sperm concentration (*p* = 0.064). Sperm motility was, however, significantly affected by infestation levels, with the least infected drones producing sperm with significantly higher total motility (99.1% vs. 83.9% and 68.2%; *p* = 0.002), progressiveness, swimming speed and kinematics (VCL, VSL, VAP; *p* < 0.001). Sperm vitality was not affected by *V. destructor* infestation (*p* = 0.559). Infestation altered sperm structure with drones from highly infected colonies producing significantly longer sperm (tail: 227 ± 1.22 *μ*m vs. 224 ± 2.31 *μ*m; total length: 236 ± 1.23 *μ*m vs. 233 ± 2.16 *μ*m; *p* < 0.001). Furthermore, significant relationships were observed between *V. destructor* infestation and sperm functionality as well as sperm structure. Colony demographics was not negatively affected by *V. destructor* infestation level, but in contrast, significant relationships were found between colony demographics and sperm motility and kinematic parameters as well as sperm structure. Findings of this study suggest that *V. destructor* infestation may hinder drone reproductive potential through subtle alterations in sperm quality, particularly sperm motility and kinematics. This study further highlights the importance of sperm quality parameters as markers of the impact of *V. destructor* infestation on honeybee drone and colony reproduction and survival.

## 1. Introduction

*V. destructor* is a near‐global ectoparasitic mite and parasite that threatens particularly managed honeybees, with devastating consequences for individual bees and colonies, if not controlled [1, 2]. Originally a parasite of the Eastern honeybee *Apis cerana (A. cerana)*, *V. destructor* shifted to the Western honeybee *Apis mellifera* (*A. mellifera*) during the first half of the twentieth century [3]. Unlike its natural host, *A. cerana*, which has co‐evolved with the mite over millennia and maintains predominantly stable infestations through several well‐established behavioural and physiological tolerance mechanisms, such as grooming behaviour, hygienic behaviour, *V. destructor*‐sensitive hygiene (uncapping and removal of mite‐infested brood), entombment of multiply‐infested drone brood and inhibition of mite fertility in worker brood [4, 5], *A. mellifera* appears to have had insufficient evolutionary time to develop effective resistance, making it highly susceptible to mite‐induced damage [3, 6]. The *V. destructor* mite parasitize honeybees by mainly feeding on their fat body, an organ that is vital for hormonal control and normal immune system functioning. The fat body also plays a critical role in vitellogenin synthesis, detoxification and energy storage, making its disruption by V. destructor particularly damaging to overall bee physiology [7, 8]. Consequently, *V. destructor* infestation compromise honeybee immunity, development, life expectancy and overall colony performance [9, 10].

The infestation and feeding of *V. destructor* mites on both immature and adult honeybees align with its reproductive and dispersal (phoretic) phases, respectively [4, 7]. In *A. mellifera*, mites primarily infest worker brood during their reproductive phase; however, *V. destructor* shows a strong preference for drone brood, a trait that is thought to have originated from its evolutionary history with the natural host, *A. cerana*, where drone brood preference also predominates [4, 6]. Furthermore, mother *V. destructor* mites particularly infest drone brood [11], and its affinity for drone brood can be attributed to several factors such as that drone larvae express much higher levels of capping pheromones and have a much longer period of *V. destructor* invasion prior to cell capping, compared with workers [2, 12, 13]. Drone brood temperature is also ideal for *V. destructor* mite development [14], and the prolonged capping phase of drone brood benefits mite reproductive potential by providing a longer time to reproduce [4, 11, 15] as drone brood is sealed for 2–3 days longer compared with worker brood [16]. The preference to infest drones may pose a risk for not only the overall health of drones but also for that of the colony because drone health is important for queen longevity, colony survival and performance [17, 18]. This risk to colony fitness is intensified by the polyandrous mating behaviour of honeybee queens, whereby a single queen mates with multiple drones during her nuptial flights and stores the pooled sperm in her spermatheca for use throughout her reproductive life, which spans over a period of 5 years [19, 20]. Genetic diversity derived from mating with multiple drones enhances colony disease resistance and overall colony strength [19]. Consequently, the quantity and quality of sperm contributed by individual drones directly determines the queen’s reproductive capacity and, by extension, the genetic diversity and performance of the colony [17, 18]. Moreover, drone health and fitness are required for successful copulation with the queen and the production of a sufficient amount of sperm, with high viability, that can successfully fertilize eggs. Therefore, it is vital to consider the impact of *V. destructor* mite infestation on drone health and its reproductive potential.

It is known that high levels of *V. destructor* mite infestation are associated with increased drone mortality and thus reduce the number of drones reaching the age of sexual maturity [21]. Furthermore, drone body mass and sexual maturity are critical parameters of drone reproductive potential. For example, drone body mass at emergence has shown to affect age of sexual maturity, sperm quality and flight fitness [19]. Sexual maturity, typically reached around 12 days post‐emergence, is a prerequisite for successful mating and queen insemination because it is only at this age that sperm migrate to the seminal vesicles, the production of mucus and maturation of mucous glands are completed and drones can copulate with a full eversion of the endophallus (average age of copulation is 21 days post‐emergence) [22, 23]. *V. destructor* mite infestation further causes weight loss during drone development, resulting in smaller adult drones [21, 24, 25]. Critically, spermatogenesis and spermiogenesis in honeybee drones are completed entirely during the larval and pupal stages of development, meaning that drones emerge as adults with a fixed complement of sperm that cannot be replenished after emergence [18]. Any disruption by *V. destructor* during brood development therefore has permanent and irreversible consequences for a drone’s reproductive potential. Regarding parameters that potentially may affect drone fertilization capability, some researchers have demonstrated reduced flight times for drones infested with *V. destructor* [26, 27] while others did not [21, 28]. *V. destructor* mite infestation has also been shown to affect drone reproductive organs such as testes, seminal vesicles and mucus gland sizes [29, 30], as well as sperm concentration [26]. However, there are others who could only find minor effects [21] or no effect on sperm concentration, volume or viability [28].

The effect of *V. destructor* mite infestation on drone sperm therefore remains inconclusive and further research is required to also investigate its effect on, apart from the above‐mentioned parameters, sperm motility and kinematic parameters, as well as sperm morphometrics, which is currently lacking. Sperm morphometrics, including measurements of sperm head, midpiece and tail dimensions, are recognised as biologically meaningful parameters because sperm tail length is directly associated with swimming velocity and fertilisation competence, and morphometric deviations may impair sperm function and reduce fertilisation success [31, 32]. Despite their critical functional importance to colony fitness, drone honeybees remain considerably understudied relative to workers, with the vast majority of *V. destructor* research focused on worker bee physiology, immunity and behaviour [4, 18]. This represents a significant knowledge gap, particularly given that drone reproductive health directly underpins queen fertility, genetic diversity and long‐term colony performance. Explicitly linking this knowledge gap to the functional importance of drones for colony fitness, understanding how *V. destructor* affects drone reproductive physiology is therefore not only relevant but essential for a comprehensive assessment of *V. destructor*’s impact on honeybee colony health and for informing bee breeding programmes aimed at improving colony resilience.

This study explored the effect of different levels of *V. destructor* mite infestation on drone reproductive physiology, specifically multiple sperm functional and structural characteristics in an understudied subspecies, *A.m. capensis,* to better understand the potential consequences on overall honeybee sperm fertilization potential. This study also considered the effect of *V. destructor* mite infestation on colony demographics as an indicator of colony performance as well as the potential subsequent effects thereof on sperm characteristics.

## 2. Materials and Methods

### 2.1. Ethics

Ethical approval for the study was obtained from the University of the Western Cape Animal ethics research committee (AREC) (AR 20/6/1).

### 2.2. Drone Collection and Colony Demographics

*A.m. capensis* drones were collected from eight different colonies originating from two different apiaries, i.e., six colonies from the one site (site A) and two colonies from another site (site B). A total of 10 drones were harvested from each individual colony for semen extraction. Drones were collecting during the mid‐morning hours before commencing on their daily flights. Both apiary sites were situated in the Stellenbosch area (Western Cape, South Africa), approximately a kilometre apart to avoid drifting of drones between colonies. The vegetation for both apiary sites was mainly fynbos plant species. Apiaries were maintained by an experienced beekeeper and according to standard apicultural practices. Although both apiaries shared similar geographic proximity and vegetation type, apiary‐specific environmental factors such as local forage availability and microclimate differences between sites cannot be fully excluded as potential sources of variability and are acknowledged as a limitation of this study. To obtain different levels of *V. destructor* infestation one of the apiaries, i.e., Site A, prior to the data collection period underwent a first‐time treatment with an acaricide (Apivar, Veto‐pharma, France) for 6 weeks during August and September 2021 according to commercial and manufacturers recommendations, while Site B colonies had never been treated with any acaricide. Formal treatment efficacy monitoring was not performed during the 6‐week treatment period. However, post‐treatment mite infestation levels recorded at the time of sampling provided indirect evidence of treatment outcome: Site A colonies recorded infestation levels below 1%–2%, while Site B colonies, which received no acaricide treatment, recorded infestation levels of approximately 10%. Amitraz‐based acaricides such as Apivar are classified as a high‐efficacy treatment (75%–99.5% mite reduction) [11, 33], and post‐treatment infestation levels below three mites per 100 bees are consistent with recommended economic and colony performance thresholds for successful treatment outcomes [11, 34], while Site B colonies had never been treated with any acaricide. Apivar contains amitraz as its active compound, a formamidine‐class acaricide that acts as an agonist on octopamine receptors in the nervous system of V. destructor, disrupting normal neurological function of the mite [35]. Although amitraz and its metabolites are known to persist in beeswax [36], Albero et al. have reported that exposure of developing brood to residual amitraz at field‐relevant concentrations did not exceed lethal thresholds [37], and Murray et al. demonstrated that in vivo Apivar treatment administered according to commercial recommendations did not significantly affect drone sperm motility, kinematics, or morphology in *A. m. capensis*, with only a significant decrease in sperm vitality observed at high in vitro concentrations [38]. Nevertheless, potential sublethal effects of residual acaricide exposure on drones collected from site A cannot be entirely excluded and are acknowledged as a limitation. The harvesting of sexually mature drones occurred between late October and beginning of December 2021, i.e., approximately 2–8 weeks after removal of the acaricide strips from Site A colonies. Collections from the two apiary sites were done on alternating days of the week and on days of collection, drones from only a single colony were collected. Sexual maturity of drones was confirmed by successful and complete eversion of the endophallus upon manual ejaculation, according to the method described by Cobey et al. [39]. Only drones that successfully everted their endophallus were included in the study. Defecating drones were also excluded to avoid the use of contaminated semen samples, resulting in a total semen sample size of 53.

Colony demographic data of colonies from which drones were harvested were collected on the same day of drone collection. Colony demographic data were determined by a trained beekeeper and assessed by scoring the following: presence of a queen, total number of frames with bees (indication of number of bees in a colony), amount of worker and drone brood present in the colony (number of brood cells in the colony), honey produced in supers and in brood (indication of amount of honey available in the colony), as well as the pollen present in brood cells (indication of the amount of pollen collected in brood cells and in the colony). Basic colony demography was collected from all colonies at each inspection period, adapted from the method described by Delaplane et al. [40]. To assess colony reproduction, the production and maintenance of worker and drone brood was used as an indicator of colony reproductive activity and food storage [40], all brood frames in a hive were assessed using a grid mentally divided into eighths and visually counted as brood, drone brood, stored pollen or stored honey. For each frame, the total count for each of the above parameters was expressed as sixteenths. Queen status for each colony was recorded, as were any noticeable pests and diseases, and frames of bees in each colony were counted using standard methods as described by Delaplane et al. [40]. All colonies had a queen present.

### 2.3. Colony *V. destructor* Infestation Level Assessment

*V. destructor* infestation levels of each colony was determined using a hot water method of Allsopp [41] whereby a small container was used to scoop up a subsample of adult bees from the frames. The use of adult bee washes at the colony level is a recognised and validated method for monitoring *V. destructor* infestation, and while it does not permit assessment of infestation at the level of individual drones, it provides a standardised and reproducible measure of colony‐level infestation pressure to which all developing brood, including drone brood, is exposed [11, 42]. The scooped bees were then transferred to a *V. destructor* mite check container (Varroa EasyCheck, Véto‐Pharma, France) and hold under running hot water until all bees were covered. The water was then decanted onto a fine sieve to count the *V. destructor* fall. The *V. destructor* infestation level of each colony was determined by counting the number of fallen mites and dividing it by the number of bees in the collected subsample to calculate percentage infestation [11, 42]. *V. destructor* infestation levels in colonies varied between 0% and 10%.

Three *V. destructor* infestation groups used for comparison were defined based on established infestation thresholds used in integrated pest management (IPM) guidelines for *V. destructor* in *A. mellifera* colonies [11]; a low infestation group (< 1%; median 0.33 (0.00%–0.40)%), representing colonies below the economic and colony performance threshold [34]; a moderate infestation group (1%–2%; median 1.07 (1.00–1.45)%), representing colonies at or approaching the intervention threshold; and a high infestation group (10%; 10.0*%* ± 0.00*%*), representing heavily infested untreated colonies with infestation levels associated with severe colony damage [3, 11]. These three groups were compared in this study.

### 2.4. Drone Semen Extraction and Sample Preparation

Drone semen samples were extracted in a nearby field laboratory in the area, transported and analysed within 1 h of extraction at the institutional laboratory. Semen samples were obtained from drones using manual ejaculation, according to the method by Cobey et al. [39], whereby the drone’s abdomen is massaged, and pressure is applied on its sides with two fingers until the abdomen hardens where after the endophallus everts with a bulb of semen at its tip. Semen was collected with a positive displacement pipette. Following ejaculation drones were weighed using a micro‐balance scale (Nimbus VR Analytical balance scale, Adam Equipment S.A. (Pty) Ltd., Kempton Park, Johannesburg, South Africa) [38]. Drone weight was recorded as dry mass, the weight of drones measured after semen extraction, immediately post‐ejaculation. Crop contents were not controlled for prior to weighing; this represents a limitation as crop contents and crop weight may vary in drones before and returning from flights, and drones inside the colony [43] and may contribute variably to total body mass. However, this approach is consistent with the method for drone weight assessment in our previous studies [38] and the effect is expected to be minimal given that drones were collected during the morning before or upon departure from the colony when crop contents are typically low [43].

Individual semen samples were diluted in 10 *μ*L Kiev solution and incubated at 37°C during transport using a mini‐dry bath (Bench mark Scientific, Whitehead Scientific (Pty) Ltd, South Africa). The preparation of samples to assess motility and kinematic parameters, sperm vitality and sperm concentration was done according to the method described by Murray et al. [38, 44]. For the preparation of each type of analyses, a sufficient part of each semen sample was used and stained in a specific ratio as described below. Briefly, for the study of motility, kinematic and vitality assessment, part of a semen sample was stained in a 1:1 ratio for 10 min with a solution made of SYBR14 (L‐7011, LTC Tech, Fairland, South Africa) and dimethyl sulfoxide (DMSO) (Sigma‐Aldrich, Johannesburg, South Africa) [44]. In preparation for the vitality assessment, a sub‐sample was removed and stained with Hoechst (33342) (L‐7011, LTC Tech, Fairland, South Africa) and a counterstain propidium iodide (PI) (L‐7011, LTC Tech, Fairland, South Africa) was used to differentiate between dead and live sperm. A Hoechst stock solution was prepared using a 50‐fold dilution in DMSO and to prepare a working solution, a five‐fold dilution was further made in Kiev buffer solution. Live sperm was stained in a 1:1 ratio with the working solution for 5 min, followed by staining with 0.5 *μ*L of PI for an additional 5 min. Staining was done in complete darkness at 37°C.

For morphology assessment, part of each semen sample was prepared according to the method of Murray (2021) [45]. Samples were stained with BrightVit (Delfran, Johannesburg, South Africa) in a 1:1 ratio for 15 min. Duplicate smears were made on glass slides and left to dry. Sperm samples were kept at 37°C during preparation and analysis to avoid functional deterioration.

### 2.5. Sperm Parameter Analyses

All sperm parameters assessed was analysed using a computer‐aided sperm analysis (CASA) system and Sperm class analyser (SCA) (version 6.5.0.44, Microptic S.L., Barcelona, Spain) as described by Murray et al. and Kotze et al., and the methods description below partly reproduces their wording [38, 44, 46]. The analyses of motility and kinematic parameters were performed by using the sample on a glass cover slide (10 *μ*m chamber depth) under a Nikon Eclipse 50i Fluorescence Microscope (IMP, Cape Town, South Africa) (fitted with a heated stage [37°C]) and a mounted Basler acA1920‐150uc USB 3.0 digital camera (Basler AG, Ahrensburg, Germany) [44]. The SCA Motility module was used to analyse sperm motility and kinematic parameters; configuration settings were Static (*μ*m/s) < 10; Medium (*μ*m/s) > 10 < 65; Rapid (*μ*m/s) > 100. For each sample, a minimum of 100 sperm was analysed and 28× objective magnification was used. In terms of motility and kinematics, the following parameters were assessed: (1) Sperm motility parameters: total motility %, progressivity (including total progressive %, non‐progressive %; medium progressive % and slow progressive %); (2) kinematic parameters: curvilinear velocity (VCL), straight‐line velocity (VSL), average path velocity (VAP), straightness (STR), linearity (LIN), wobble (WOB,) beat cross frequency (BCF), the amplitude of lateral head displacement (ALH) and dance (DNC) [38].

As for motility analyses, sperm vitality was analysed using fluorescence microscopy, with the Vitality module of SCA, which distinguish between viable sperm cells (blue) and non‐viable cells (red) [38]. A smaller number of drones’ samples were analysed in the lowest and highest infestation group due to some of the samples drying out before analyses could be performed. The number of drone samples analysed for vitality as per infestation group was: < 1%: *n* = 9; 1*%*–2*%*: *n* = 19; 10%: *n* = 9.

Sperm concentration (expressed as million/*μ*L) was determined by using the SYBR14 stained sample. As semen volume was not measured in this study (Section 2.4), sperm concentration was assessed on the basis of count per unit volume of the diluted sample rather than total sperm count per ejaculate. In the Morphology module of SCA, a digital Makler chamber was used to view the captured fields. The number of sperm in 10 chambers were counted, multiplied by the dilution factor and divided by 1000 [38, 44]. This method is consistent with that described by Murray et al. [38, 44] and provides a standardised measure of sperm density per unit volume of the diluted sample, independent of total ejaculate volume. Comparisons between infestation groups are therefore based on equivalent sample preparation volumes across all drones, ensuring methodological consistency.

Analysis of morphology slides was done using the SCA Morphology module with bright field microscopy on a Nikon Eclipse 50i microscope and mounted digital camera (Basler ace acA1300‐200*μ*c colour USB 3.0; Microptic S.L., Barcelona, Spain). A ×40 objective was used to measure the sperm head length, and a ×20 objective was used to measure sperm tail and total sperm length. A minimum of 50 individual sperm was measured per drone sample. As a result of semen samples that dried out before preparation for morphology assessment, only a small number of drones from each of the infestation groups could be analysed further, i.e., two drones per group, and the number of samples for each of the two drones that were analysed for morphology as per infestation group was as follows: < 1%, *n* = 8; 1%–2%, *n* = 12 and 10%, *n* = 9.

Semen volume was not assessed in this study.

### 2.6. Statistical Analyses

MedCalc Statistical Software version 20.111 (MedCalc Software Ltd, Ostend, Belgium) was used to perform statistical analyses of data. To determine the distribution of data, the Shapiro–Wilk test for normal distribution was deployed. Comparisons between the *V. destructor* infestation groups in terms of drone weight, sperm characteristics (e.g., motility, kinematics, vitality, concentration), sperm morphometrics and colony demographics were done using either a one‐way analyses of variance (ANOVA) test for normally distributed data and Kruskal–Wallis test for data that was not normally distributed. Additionally, data analysed with the ANOVA test was further subjected to the Levene’s test for equality, and data that resulted in a *p* value of <0.05 was analysed using the Kruskal–Wallis test. Parametric data is reported as mean and standard deviation (SD), while non‐parametric data is reported as the median and 25%–75% percentiles.

Correlations between *V. destructor* infestation and sperm characteristics as well as colony demographics were tested using the Pearson’s correlation test (the Pearson’s coefficient was reported as *r*) in case of normally distributed data, and the Spearman rank correlation test in case of data that was not normally distributed (the Spearman coefficient was reported as *ρ* [rho]).

Multivariate visualisation methods, i.e., star glyphs and sunray plots, were constructed to highlight the differences among the three infestation groups (Statgraphics, Centurion software, Version 19, The Plains, Virginia) for motility and kinematic parameters. In the case of the star glyphs and sunray plots, each sperm parameter (variable) is assigned to a spoke or axis radiating from the central point. The key glyph provides the ranges of values of sperm parameters along each axis. In the case of the star glyph, the length of the axis represents the value of the parameter, and the end points of all the parameters form the star shape when they connect. In the case of the sunray plots, line segments, colours and shading provide an angular arrangement. Together, the elements on the graphs represent specific sperm parameters or its values as constructed by Statgraphics [47].

## 3. Results

Colonies from Site A (treated with Apivar) recorded post‐treatment infestation levels of < 1% (*n* = 4 colonies) and 1%–2% (*n* = 2 colonies), while colonies from Site B (untreated) recorded infestation levels of 10% (*n* = 2 colonies). Because of the unequal and small number of colonies per site, formal statistical comparison of infestation levels between sites was not performed; differences in infestation levels are therefore reported descriptively.

### 3.1. Effect of V. destructor Infestation on Drone Weight and Sperm Concentration

Although not statistically significantly, drones exposed to lower *V. destructor* infestation levels weighed 11% more (< 1%: 172 (161–179) mg; 1%–2%: 172 (162–178) mg) compared with drones from highly infested colonies (10%: 153 (141–163) mg) (*p* = 0.123). The sperm concentration also did not differ among the groups and was very similar between the lowest (2.28 (1.5–3.07) × 10^6^ mil/*μ*L) and highest *V. destructor* infestation (2.24 (1.29–3.51) × 10^6^ mil/*μ*L) groups, but lower in the 1%–2% group (1.44 (1.20–2.00) × 10^6^ mil/*μ*L) (*p* = 0.064). Furthermore, no correlation was found between sperm concentration and the percentage of *V. destructor* infestation (*p* = 0.664).

### 3.2. Effect of V. destructor Infestation on Motility and Kinematic Parameters

**Figure 1:**
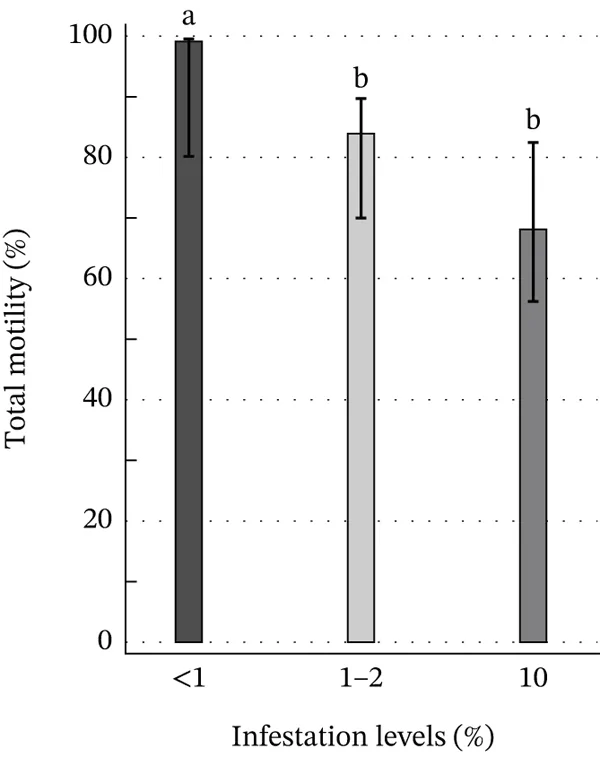
Effect of different levels of *V. destructor* mite infestation on sperm total motility percentage. Data is presented as median (25th,75th percentiles). Error bars with different letters indicate significant differences among the three different *V. destructor* mite infestation groups, i.e., < 1% (drones *n* = 25; colonies *n* = 4), 1%–2% (drones *n* = 19; colonies *n* = 2) and 10% (drones *n* = 9; colonies *n* = 2).

The majority of sperm were non‐progressive (> 60%) and slow swimming (> 60%) (Table S1). Sperm motility percentage decreased with increasing infestation, with drones from colonies with the lowest level of *V. destructor* infestation (< 1%) producing sperm with a significantly higher motility percentage (99.1 (80.1–99.5) %) compared with drones from more infested colonies (1%–2%:83.9 (69.9–89.7)%; 10%: 68.2 (56.2–83.5)%) (*p* = 0.002) (Figure 1; Table S1). It should be noted that the percentage of sperm total motility comprises the percentage progressive, (including total‐, rapid‐ and medium‐progressive) and non‐progressive sperm. Non‐progressive sperm was significantly lower in the highest infestation group (10%:67.2 (56.1–75.6)) (*p* = 0.055) compared with the lower infestation groups (< 1%: 79.8 (72.2–85.1), 1%–2%:79.8 (69.6–84.1)). The relationship between *V. destructor* infestation and sperm motility parameters were further confirmed with significant, but weak negative correlations between *V. destructor* infestation and most motility parameters, except for non‐progressive and slow swimming sperm for which no significant correlations were found (Table S2). Sperm from the lowest infestation group also had significantly more progressive (total‐, rapid‐ and medium progressive) and faster swimming sperm (rapid and medium) compared with the other groups (*p* < 0.001), however, these parameters constitute only a small percentage of the total sperm population.

**Figure 2:**
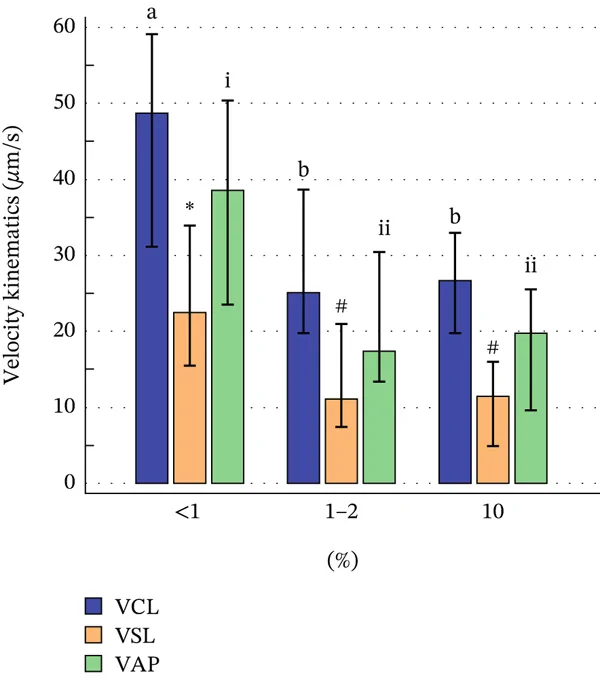
Effect of different levels of *V. destructor* infestation on sperm velocity kinematics. Data is presented as median (25th,75th percentiles). Error bars with different letter, symbol or roman numerals indicate significant differences among the three different *V. destructor* infestation groups, i.e., < 1% (drones *n* = 25; colonies *n* = 4), 1%–2% (drones *n* = 19; colonies *n* = 2) and 10% (drones *n* = 9; colonies *n* = 2) for the three variables VCL, VSL and VAP. VAP, average path velocity; VCL, curvilinear velocity; VSL, straight‐line velocity.

Similar to the motility parameter results, the least infested drones had sperm with significantly higher velocity kinematic speeds for VCL, VSL and VAP, compared with the higher infestation groups (*p* < 0.001; *p* = 0.001; *p* < 0.001) (Figure 2; Table S1). The least infested group also presented with significantly higher percentages for the velocity ratio kinematics LIN (*p* = 0.005), STR (*p* = 0.014) and WOB (*p* = 0.003), which is expected as VSL, VCL and VAP are used for the calculation thereof. Additionally, parameters representing sperm wobble characteristics, i.e., ALH (*p* = 0.001) and BCF (*p* = 0.001), were also significantly higher in the least infested group compared with the other groups. As for motility parameters, significant but weak correlations were shown between *V. destructor* infestation and kinematic parameters, except for STR (Table S2).

Considering that majority of sperm in all groups, and in honeybee semen in general, are slow swimming, we further explored the effect of *V. destructor* infestation on the velocity kinematic parameters (VCL, VSL and VAP) of the slow swimming sperm subpopulation. For all three these parameters, the least infested group demonstrated significantly higher values compared with the other groups with a higher infestation (VCL, *p* < 0.001; VSL, *p* = 0.001; VAP, *p* < 0.001) (Table S3).

**Figure 3:**
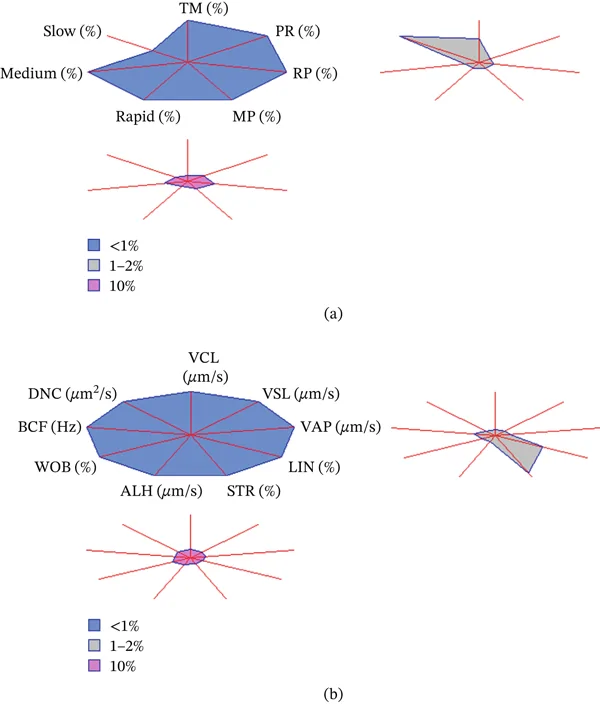
Star glyphs and sunray plots presenting differences in (a) sperm motility parameters and (b) sperm kinematic parameters among three infestation groups (blue = <1*%*; grey = 1*%*–2*%*; purple = 10*%*). The plots distinguish cases that have similar values of the input variables used. The key glyph indicates the variables used, i.e., seven variables in (a) and nine variables in (b). ALH, amplitude of lateral head displacement; BCF, beat cross frequency; DNC, dance; LIN, linearity; MP, medium progressive; PR, progressive; RP, rapid progressive; STR, straightness; TM, total motility; VAP, average path velocity; VCL, curvilinear velocity; VSL, straight‐line velocity; WOB, wobble.

Weak to stronger correlations were noted between sperm concentration and majority of sperm motility and kinematic parameters (except for slow swimming sperm) (Table S4). Lastly, star glyph and sunray plots presented in Figure 3 below compress the described motility (Figure 3a) and kinematic (Figure 3b) data to illustrate the differences among the three infestation groups.

### 3.3. Effect of V. destructor Infestation on Sperm Vitality

Sperm vitality percentage was not affected by level of *V. destructor* infestation (*p* = 0.559). The overall vitality was relatively high. Interestingly, vitality seemed to have increased as *V. destructor* infestation level increased (Table S1). Vitality, however, was lower than total motility, i.e., 23% and 6% lower in the < 1% and 1%–2% infestation groups, respectively, but in contrast, vitality was 17% higher than total motility in the 10% infestation group. Vitality results should be similar or greater than the total motility results, since motility is a subset of the total viable sperm [48, 49]. The discrepancy could be ascribed to the fact that vitality was assessed an hour after semen collection, resulting in stained samples that started to dry out with the dehydration killing sperm.

### 3.4. Effect of V. destructor Infestation on Sperm Morphometrics

Increased *V. destructor* mite infestation exposure appeared to result in increased sperm length, with the highest level of *V. destructor* infestation resulting in significantly longer sperm tails (227 ± 1.22 *μ*m) (*p* < 0.001) and total sperm lengths (236 ± 1.23 *μ*m) ($\underset{\_}{p}<0.001$) (Table 1). Despite this significance, the percentage difference in terms of *μ*m between the lower and higher infestation groups for sperm length and total sperm length is actually very small, 1.3%. *V. destructor* infestation level did not affect sperm head length nor the head:tail ratio. However, significantly strong, positive correlations were also found between *V. destructor* infestation and the sperm tail (*r* = 0.709; *p* < 0.001) and total sperm lengths (*r* = 0.716; *p* < 0.001) (Table S5).

**Table 1:** Comparison of the effect of *V. destructor* infestation on sperm component lengths.

Sperm component	*V. destructor* infestation			*p*value	Test statistic
	< 1% (*n* = 8)	1‐2% (*n* = 12)	10% (*n* = 9)		
Sperm head Length (*μ*m)	9.00 ± 0.22	8.91 ± 0.40	9.07 ± 0.00	0.582	1.080
Sperm tail length (*μ*m)	224 ± 2.31^a^	224 ± 1.79^a^	227 ± 1.22^b^	< 0.001	12.52
Sperm total length (*μ*m)	233 ± 2.16^a^	233 ± 1.97^a^	236 ± 1.23^b^	< 0.001	12.73
Head:tail ratio	0.04 ± 0.00	0.04 ± 0.00	0.04 ± 0.00	0.795	0.231

*Note:* Data is reported as mean and standard deviation (mean ± SD). ^ab^ Means with different superscript letters in the same row denote significant differences. (drones *n* = 8; colonies *n* = 4), 1%–2% (drones *n* = 12; colonies *n* = 2) and 10% (drones *n* = 9; colonies *n* = 2).

Regarding the relationship between structure and function, only a weak significant negative correlation was found between the percentage of slow swimming sperm and sperm length (*ρ* = −0.416; *p* = 0.028) as well as total sperm length (*ρ* = −0.424; *p* = 0.024) (the percentage of slow swimming sperm increased as the sperm length decreased). Thus, the lower infestation groups (< 1% and 1%–2%) had a higher percentage of slow swimming sperm, and shorter sperm compared with the sperm obtained from colonies with the highest infestation level. There were no significant correlations between drone weight and sperm component lengths.

### 3.5. Effect of V. destructor Infestation on Colony Demographics

Colony demographic data were collected on the same day as drone collection, at which point *V. destructor* infestation levels were recorded as follows: low infestation group (< 1%; median 0.33 (0.00%–0.40)%, *n* = 6 colonies); moderate infestation group (1%–2%; median 1.07 (1.00–1.45)%, *n* = 2 colonies); and high infestation group (10%; 10.0 ± 0.00*%*, *n* = 2 colonies). *V. destructor* infestation level did not significantly affect any colony demographic parameters (Table S6). Although no statistically significant differences were detected, a trend was observed whereby colonies with higher infestation levels tended to have fewer frames with bees, less drone brood and less honey; however, these observed trends should not be interpreted as effects given the absence of statistical significance and the small number of colonies assessed. There were no correlations between *V. destructor* infestation and demographic variables of colonies from which drone samples were collected (*n* = 8) (Table S7).

On the other hand, the colony demographics appeared to relate to sperm functional and structural characteristics. Weak correlations were found between brood pollen, amount of worker brood, total honey and sperm motility and kinematic parameters (Table S8), but weak and moderate correlations were noted between these demographic parameters and sperm morphometrics (Table S9). Statistically significant positive correlations were found between brood pollen and worker brood in colonies and sperm head length (r = 0.524; *p* = 0.004; *n* = 29). A significant negative correlation was found between total honey and sperm head length (r = −0.404; *p* = 0.030). Stronger negative correlations were found between total honey and sperm tail length (r = −0.618; *p* < 0.001) and total sperm length (r = −0.647; *p* < 0.001).

## 4. Discussion

In agreement with reports on other species, *A.m. capensis* drone weight, as a measure of body size, was negatively affected by *V. destructor* infestation. However, the effect in this study appears to be less than on *Apis mellifera scutellata* drones. In the latter sub‐species, Strauss et al. [25] observed a weight loss of 3.7% for one mite and 8.6% for ≥ 4 mites/drone cell. However, a different method was used compared with this study, i.e., mite infestation was done by counting mites in emerging drone’s cells and recording the weight of these emerging drones [25], rather than using colony mite infestation levels. Furthermore, Duay et al. [24] reported an even greater weight reduction of 11% for one to three mites and 18.3% for ≥ 4 mites/drone cell in *Apis mellifera carnica*. Omar [30] also reported a larger weight loss with an approximately 16%–17% weight loss for 10‐ and 17‐day old *Apis mellifera carnica* (Carniolan) drones from colonies with a 7%–10% *V. destructor* infestation level, respectively. It has been shown that the weight loss observed in drones may be partly ascribed to the loss of water because of *V. destructor* parasitism [25]. The findings on drone body weight underscore the suggestion by Strauss et al. [25] that South African honeybee species are more tolerant to *V. destructor* mite infestation. It should be noted, however, that the observed difference in drone weight between infestation groups in this study was not statistically significant, and alternative explanations for the trend toward reduced body mass in highly infested colonies cannot be excluded. Reduced drone weight in high‐infestation colonies may also reflect indirect nutritional stress arising from colony health decline, given that *V. destructor* feeding depletes fat body tissue, a critical organ for immune function, nutrient storage and overall bee fitness [1, 7] in addition to the direct effects of mite parasitism on individual drone brood. The relatively smaller weight effect observed in *A. m. capensis* is consistent with the reported tolerance mechanisms in southern African honeybee subspecies, including shorter brood capping periods that limit mite reproduction opportunities and higher frequencies of hygienic behaviour and grooming alleles compared with European subspecies [41].

Furthermore, in agreement with Collins and Pettis [28] reporting on moderately infested drones (the number of mites or infestation level were not mentioned), sperm concentration in the most and least infested groups of this study was similar, despite differences in drone weight. Rinderer et al. [21] also observed minor effects of *V. destructor* infestation (1% drone brood infestation and one to five mites per drone cell) on drone sperm count. In contrast, Duay et al. [26] found a reduction in the number of sperm as mite numbers increased (24% reduction for one mite and 45% reduction for two mites/drone brood cell) (drone weight was not reported). Schneider [29] further reported a 50% decrease in sperm numbers for drones infested by more than three mites, and Bubalo et al. [50] have shown that lightly infested drones had higher sperm numbers compared with heavily infested drones. Discrepancy in results could be attributed to the differences in methods to determine infestation levels.

Considering sperm functionality (motility and kinematic parameters), the findings suggest that drones exposed to very low *V. destructor* mite infestation levels (< 1%) have the potential to produce sperm of better functional quality. Although sperm motility is also regarded as an indicator of honeybee sperm quality, it is only representative of the entire sperm population and may not reveal the true effect of factors that sperm are exposed to [51]. Therefore, the analysis of sperm subpopulations is important. In agreement with previous work on *A.m. capensis* sperm subpopulations [44], slow swimming sperm made up most of the sperm population. Thus, to determine the true effect of *V. destructor* infestation on drone sperm, we consider it of value to report on the velocity kinematics of slow swimming sperm. It is important to distinguish between sperm motility percentage, referring to the proportion of sperm exhibiting any movement, and velocity kinematic parameters, which quantify the speed and trajectory of individual sperm movement (VCL, VSL and VAP). These represent distinct and complementary aspects of sperm function. In this study, both total motility percentage and velocity kinematic parameters were significantly lower in drones from more infested colonies, indicating that *V. destructor* infestation affects both the proportion of motile sperm and the swimming speed of individual sperm cells. Furthermore, the velocity kinematic parameters of the slow‐swimming subpopulation were also significantly reduced with increasing infestation, suggesting that the effect of *V. destructor* infestation on sperm motility extends beyond the overall population to the dominant sperm subpopulation. Whether a *V. destructor* infestation level of < 1% represents the threshold at which sperm functionality begins to be negatively impacted requires further investigation with larger sample sizes. Notably, the < 1% threshold aligns with established IPM guidelines as the level below which colony performance is considered uncompromised [11, 34], making this an ecologically meaningful reference point for future studies.

Sperm vitality was not affected by *V. destructor* infestation in this study and the overall sperm vitality exceeded 75% in all infestation groups. According to Collins [52] sperm viability above 50% is sufficient for a queen to lay fertilized eggs. The vitality percentages recorded in this study are within ranges reported by Pettis et al. for healthy honeybee drones, where naturally‐mated queen spermathecae contained sperm with viability of 57%–92% depending on colony health status [17]. However, the vitality sample sizes in this study were small (< 1%: *n* = 9; 1%–2%: *n* = 19; 10%: *n* = 9), limiting statistical power to detect potential group differences. The discrepancy observed between vitality and total motility values, notably that vitality was lower than motility in the <1% and 1%–2% groups, is likely attributable to the methodological constraint that vitality was assessed approximately 1 h after semen collection, during which time sample dehydration may have reduced the proportion of viable sperm detected. In the context of queen polyandry, where queens mate with an average of 12 drones and store 5–7 million sperm in the spermatheca [53], the reproductive contribution of individual drones is determined not only by sperm viability but by sperm competitive ability and longevity in storage [54–56]. Whether the observed differences in sperm motility and kinematics associated with *V. destructor* infestation levels translate into reduced fertilization potential or diminished paternity share under competitive sperm storage conditions remains an important gap that warrants investigation.

Additionally, the production of sperm with longer tails from drones exposed to the highest infestation level and strong correlations between *V. destructor* infestation level and sperm tail length, suggests that *V. destructor* mite parasitism may alter sperm structural development. Del Cacho et al. [57] suggested that glycoproteins in the plasma membrane of insect sperm are important for sperm maturation and have demonstrated that *V. destructor* infestation of drones negatively impact the number of glycoproteins (reduced number of lectin binding sites) in the acrosomal region of sperm. Such effects on the tail region of insect sperm have not been reported. Drone sperm tail elongation has been noticed between the beginning and end of seasons [31]. However, because the drones in this study were all collected within 2 months in the summertime, this seems unlikely to have played a role. It should also be acknowledged that since drones in the high infestation group (10%) were collected exclusively from Site B colonies, and drones in the low infestation groups (< 1% and 1%–2%) were collected from Site A colonies, genetic differences between colonies at the two sites cannot be excluded as a contributing factor to the observed morphometric differences, in addition to infestation level effects. The two apiaries were geographically proximate but represented distinct colony populations, and inter‐colony genetic variation in sperm morphometrics is a recognised source of variation in honeybees [31, 58]. Future studies should use a more balanced sampling design across sites and infestation levels to disentangle the effects of infestation from genetic background.

Lastly, although colony demographics were not affected by *V. destructor* infestation, relationships were found between colony demographics and sperm motility, kinematic and structural parameters. The observed correlations between colony nutritional parameters (brood pollen, worker brood, total honey) and sperm morphometrics suggest that colony‐level nutritional status may indirectly influence drone sperm development. Drones depend entirely on worker‐prepared food during larval development [43], and colony nutritional resources, reflected by honey stores and pollen availability, may affect the quality of food provisioned to developing drone larvae. However, given the small number of colonies assessed, formal testing of these relationships using generalised linear mixed‐effects models incorporating colony as a random effect and infestation level and nutritional parameters as fixed effects was not possible in this study. Such an analytical approach would substantially increase statistical power and allow more integrative interpretation of these associations and is recommended for future studies with larger sample sizes. Rinderer et al. [21] recommends the assessment of *V. destructor* infestation of drone brood to help determine drone abundance in a colony and indicated that colonies with the highest *V. destructor* infestation (58.6% of drones infested) may appear to have the most drone brood but suffer a high drone mortality rate before reaching sexual maturity. However, the amount of drone brood in the highly infested colonies were least in this study but was far less compared with the study by Rinderer et al. [21].

The limitations of the study include the cross‐sectional nature of the study, restricted to one season and the use of small sample sizes for drones and colonies. Sample sizes also differed between analyses: motility and vitality assessments were based on a larger number of drones, whereas morphology analyses were limited because a subset of samples dried out. Additionally, although this study used a colony‐level adult bee wash infestation assessment, a standardised and validated method [11, 42], which provides a reproducible measure of infestation pressure experienced by all developing brood in a colony, this method does not allow confirmation of infestation at the level of individual drones, which represents a recognised limitation when interpreting individual‐level reproductive outcomes. The inability to confirm whether assessed drones were individually parasitised is acknowledged as a constraint on the strength of causal inference in this study. Furthermore, although brood exposure at field‐relevant amitraz concentrations does not seem to pose a threat to drone sperm quality in *A. m. capensis* [38], potential sublethal effects of residual acaricide on drones from the treated Site A cannot be entirely excluded. Considering these limitations, extrapolation of results should be done with caution. The single‐season design reflects the constraints of the available dataset and the cross‐sectional scope of this study and highlights the need for longitudinal studies across multiple seasons and with greater colony replication to confirm the trends observed here.

## 5. Conclusion

Notwithstanding the study limitations, the findings of this study have applied relevance for beekeeping, selective breeding and the management of natural honeybee populations. The association between elevated *V. destructor* infestation and reduced drone sperm motility and velocity kinematics suggests that uncontrolled mite infestations may compromise the reproductive quality of drones in managed colonies, with potential downstream consequences for queen insemination success and colony genetic diversity. Given that queen polyandry and the associated genetic diversity of worker populations are critical determinants of colony health and resilience [59], maintaining low *V. destructor* infestation levels through effective IPM may be important not only for worker health but for preserving drone reproductive integrity. For selective breeding programs targeting *V. destructor*‐tolerant *A. m. capensis* colonies, drone reproductive quality under varying infestation pressures represents an underexplored selection criterion. Furthermore, in natural *A. m. capensis* populations where *V. destructor* is present without systematic treatment, the cumulative effects of sustained infestation on drone sperm quality and colony reproductive fitness warrant further investigation.

## Funding

This study was supported by National Research Foundation South Africa, TTK210303588672.

## Disclosure

AI technology, i.e., ChatGPT was used to assist with coherency and flow of information in the Discussion section; Claude (Anthropic) assisted with overall structuring and refining sections.

## Conflicts of Interest

The authors declare no conflicts of interest.

## Supporting information


**Supporting Information** Additional supporting information can be found online in the Supporting Information section. Table S1: Effect of *V. destructor* infestation level on sperm motility and kinematic parameters and sperm vitality. Table S2: Correlations between *V. destructor* infestation percentage and sperm motility and kinematic parameters. Table S3: Effect of *V. destructor* infestation on the sperm subpopulation velocity kinematics. Table S4: Correlations between sperm concentration and sperm motility and kinematic parameters. Table S5: Correlations between *V. destructor* infestation percentage and sperm morphometrics. Table S6: Effect of *V. destructor* infestation on colony demographics. Table S7: Correlations between *V. destructor* infestation and demographics of colonies from which drones were harvested. Table S8: Spearman’s Rank Correlations between colony demographics and sperm motility and kinematic parameters. Table S9: Pearson’s correlations between colony demographics versus sperm morphometrics.

## Data Availability

Data can be requested from the corresponding author.
