# Supplementary material for: Effects of Varroa destructor on Apis mellifera capensis Drone Sperm Quality Characteristics and Colony Demographics
Source: J Parasitol Res. 2026 Sep 1;2026:8285152. doi: 10.1155/japr/8285152 (PMC13532402; doi:10.1155/japr/8285152)
Supplement: Supplementary file 1 [file JAPR-2026-8285152-s001.docx]

**Supplementary material**

**Supplementary Table 1.** Effect of *V.destructor* infestation level on sperm motility and kinematic parameters and sperm vitality.

|  | **Infest <1 %**  (n=25) | **Infest 1-2 %** (n=19) | **Infest 10 %**  (n=9) | **P Value** | **Test statistic** |
| --- | --- | --- | --- | --- | --- |
| **Motility parameters** | | | | | |
| TM (%) | 99.1 (80.1-99.5)^a^ | 83.9 (69.9-89.7)^b^ | 68.2 (56.2-83.5)^b^ | 0.002 | 12.77 |
| PR (%) | 9.62 (4.16-18.7)^a^ | 0.60 (0.23-5.91)^b^ | 1.24 (0.09-4.54)^b^ | <0.001 | 15.39 |
| NP (%) | 79.8 (72.2-85.1) | 79.8 (69.6-84.1) | 67.2 (56.1-75.6) | 0.055 | 5.807 |
| RP (%) | 0.91 (0.56-1.92)^a^ | 0.00 (0.00-0.35)^b^ | 0.14 (0.00-0.50)^b^ | <0.001 | 17.93 |
| MP (%) | 8.72 (3.47-17.2)^a^ | 0.60 (0.23-5.41)^b^ | 1.24 (0.09-4.24)^b^ | 0.001 | 1.964 |
| Rapid (%) | 2.33 (0.96-7.54)^a^ | 0.14 (0.00-0.57)^b^ | 0.14 (0.00-1.01)^b^ | <0.001 | 17.35 |
| Medium (%) | 16.2 (5.56-31.0)^a^ | 1.37 (0.54-7.43)^b^ | 2.90 (0.93-6.87)^b^ | <0.001 | 15.45 |
| Slow (%) | 65.9 ± 13.7 | 73.2 ± 10.8 | 62.0 ± 10.6 | 0.049 | 3.201 |
| **Kinematic parameters** | | | | | |
| VCL (µm/s) | 48.7 (31.1-59.1)^ab^ | 25.1 (19.7-38.7)^b^ | 26.7 (19.8-33.0)^b^ | <0.001 | 16.22 |
| VSL (µm/s) | 22.5 (15.5-34.0)^ab^ | 11.1 (7.41-21.0)^b^ | 11.5 (4.89-16.0)^b^ | 0.001 | 13.92 |
| VAP (µm/s) | 37.6 ± 15.5^ab^ | 20.6 ± 10.9^b^ | 20.7 ± 14.3^b^ | <0.001 | 10.07 |
| LIN (%) | 46.9 (37.9-55.8)^ab^ | 38.1 (32.0-46.2)^b^ | 31.2 (21.2-37.5)^b^ | 0.005 | 10.76 |
| STR (%) | 57.3 (50.9-65.0)^a^ | 55.9 (50.3-57.7)^a^ | 44.0 (37.0-49.5)^b^ | 0.014 | 8.469 |
| ALH (µm/s) | 1.19 (0.99-1.46)^ab^ | 0.89 (0.83-1.07)^b^ | 0.90 (0.79-1.00)^b^ | 0.001 | 13.16 |
| WOB (%) | 78.8 (67.8-82.1)^ab^ | 64.2 (56.6-72.5)^b^ | 65.0 (46.7-71.0)^b^ | 0.003 | 11.73 |
| BCF (Hz) | 7.76 (4.85-10.6)^ab^ | 4.36 (3.33-6.54)^b^ | 4.10 (2.26-5.49)^b^ | 0.001 | 14.45 |
| DNC (µm^2^/s) | 111 (57.4-179)^ab^ | 44.1 (31.5-83.2)^b^ | 46.9 (31.1-66.7)^b^ | 0.001 | 14.55 |
| **Vitality** | | | | | |
|  | **Infest <1 %**  (n=9) | **Infest 1-2 %** (n=19) | **Infest 10 %**  (n=9) | **P Value** | **Test statistic** |
| Vitality % | 76.2 ± 10.4 | 78.8 ± 12.0 | 82.2 ± 11.9 | 0.559 | 0.592 |

Parametric data is presented as mean and standard deviation (SD), while non-parametric data is presented as median and 25 to 75 percentiles. ^ab^ Means or medians with different superscript letters in the same row indicate significant differences. TM, total motility; PR, progressive; NP, non-progressive; RP, rapid progressive; MP, medium progressive; VCL, curvilinear velocity; VSL, straight-line velocity; VAP, average path velocity; LIN, linearity; STR, straightness; ALH, amplitude of lateral head displacement; WOB, wobble; BCF, beat cross frequency; DNC, dance.

**Supplementary Table 2.** Correlations between *V.destructor* infestation percentage and sperm motility and kinematic parameters.

| ***V.destructor*** **Infestation (%) *versus*** | **Spearman’s rank correlation**  *ρ* **(rho)** | **P value** |
| --- | --- | --- |
| **Motility parameters** (n=53) | | |
| TM (%) | -0.375 | 0.007 |
| Progressive (%) | -0.368 | 0.008 |
| RP (%) | -0.402 | 0.004 |
| MP (%) | -0.356 | 0.010 |
| NP (%) | -0.223 | 0.107 |
| Rapid (%) | -0.410 | 0.003 |
| Medium (%) | -0.352 | 0.011 |
| Slow (%) | -0.065 | 0.639 |
| **Kinematic parameters** (n=53) | | |
| VCL (µm/s) | -0.382 | 0.006 |
| VSL (µm/s) | -0.338 | 0.015 |
| VAP (µm/s) | -0.356 | 0.010 |
| LIN (%) | -0.275 | 0.047 |
| STR (%) | -0.209 | 0.133 |
| WOB (%) | -0.288 | 0.038 |
| ALH (µm/s) | -0.359 | 0.010 |
| BCF (Hz) | -0.357 | 0.010 |

**Supplementary Table 3:** Effect of *V.destructor* infestation on the sperm subpopulation velocity kinematics.

| **Sub-population** | **Slow swimming sperm** | | | **P value** |
| --- | --- | --- | --- | --- |
| ***V.destructor* infestation** | **<1%**  (n=25**)** | **1-2%**  (n=19) | **10%**  (n=9) |  |
| **Velocity kinematic parameters** | | | | |
| VCL | 35.0 (27.7-40.2)^a^ | 25.3 (23.4-30.2)^b^ | 25.0 (23.2-29.6)^b^ | <0.001 |
| VSL | 19.5 (12.5-24.7)^a^ | 11.5 (8.23-16.6)^b^ | 10.2 (8.97-13.2)^b^ | 0.001 |
| VAP | 27.0 ± 7.92^a^ | 18.2 ± 5.45^b^ | 18.5 ± 6.73^b^ | <0.001 |

Parametric data is presented as mean and standard deviation (mean ± SD), while non-parametric data is presented as median and 25 to 75 percentiles. ^ab^ Means or medians with different superscript letters in the same row indicate significant differences. VCL, curvilinear velocity; VSL, straight-line velocity; VAP, average path velocity.

**Supplementary Table 4:** Correlations between sperm concentration and sperm motility and kinematic parameters.

| **Sperm Concentration *versus*** | **Spearman’s rank correlation**  *ρ* **(rho)** | **P value** |
| --- | --- | --- |
| **Motility parameters** (n=53) | | |
| TM (%) | 0.466 | <0.001 |
| PR (%) | 0.506 | <0.001 |
| RP (%) | 0.482 | <0.001 |
| MP (%) | 0.509 | <0.001 |
| NP (%) | 0.385 | 0.004 |
| Rapid (%) | 0.511 | <0.001 |
| Medium (%) | 0.510 | <0.001 |
| Slow (%) | 0.161 | 0.249 |
| **Kinematic parameters** (n=53) | | |
| VCL (µm/s) | 0.528 | <0.001 |
| VSL (µm/s) | 0.519 | <0.001 |
| VAP (µm/s) | 0.548 | <0.001 |
| LIN (%) | 0.470 | <0.001 |
| STR (%) | 0.361 | 0.001 |
| WOB (%) | 0.602 | <0.001 |
| ALH (µm/s) | 0.541 | <0.001 |
| BCF (Hz) | 0.543 | <0.001 |

**Supplementary Table 5:** Correlations between *V.destructor* infestation percentage and sperm morphometrics.

| ***V.destructor* infestation (%) *versus*** | **Pearson’s correlation coefficient (*r)*** | **P value** |
| --- | --- | --- |
| Head length (µm) | 0.208 | 0.280 |
| Tail length (µm) | 0.709 | <0.001 |
| Total sperm length (µm) | 0.716 | <0.001 |
| Head:Tail ratio | -0,039 | 0.842 |

**Supplementary Table 6**. Effect of *V.destructor* infestation on colony demographics.

| **Colony demographics** | ***V.destructor* infestation** | | | **P Value** | **Test statistic** |
| --- | --- | --- | --- | --- | --- |
|  | **<1 %**  (n=3) | **1-2 %**  (n=3) | **10 %**  (n=2) |  |  |
| Total amount of frames with bees | 17.0 ± 2.65 | 15.7 ± 6.66 | 11.5 ± 4.95 | 0.522 | 0.741 |
| Brood pollen collected | 3.88 ± 1.65 | 4.31 ± 0.86 | 4.44 ± 0.44 | 0.854 | 0.163 |
| Amount of worker brood | 5.21 ± 0.75 | 4.31 ± 0.86 | 4.44 ± 0.44 | 0.372 | 1.211 |
| Amount of drone brood | 0.38 ± 0.33 | 0.42 ± 0.31 | 0.22 ± 0.13 | 0.763 | 0.286 |
| Honey produced (supers and brood) | 7.73 ± 2.20 | 5.33 ± 4.43 | 3.33 ± 1.65 | 0.385 | 1.161 |

Parametric data is presented as mean and standard deviation (SD).

**Supplementary Table 7**: Correlations between *V.destructor* infestation and demographics of colonies from which drones were harvested.

| ***V.destructor* infestation (%) *versus*** | **Spearman’s rank correlation**  *ρ* **(rho)** | **P value** |
| --- | --- | --- |
| Total frames with bees | -0.293 | 0.482 |
| Amount of worker brood | -0.259 | 0.536 |
| Amount of drone brood | -0.173 | 0.682 |
| Brood pollen collected | 0.223 | 0.596 |
| Total honey produced (supers and brood) | -0.515 | 0.192 |

**Supplementary Table 8:** Spearman’s Rank Correlations between colony demographics and sperm motility and kinematic parameters.

|  | **Brood pollen collected *versus*** | | **Amount of worker brood *versus*** | | **Total honey produced *versus*** | |
| --- | --- | --- | --- | --- | --- | --- |
|  | *ρ* **(rho)** | **P value** | *ρ* **(rho)** | **P value** | *ρ* **(rho)** | **P value** |
| **Motility parameters** (n=53) |  |  |  |  |  |  |
| TM (%) | 0.244 | 0.079 | 0.127 | 0.361 | 0.347 | 0.012 |
| Progressive (%) | 0.264 | 0.057 | 0.269 | 0.053 | 0.298 | 0.032 |
| RP (%) | 0.322 | 0.020 | 0.322 | 0.020 | 0.328 | 0.018 |
| MP (%) | 0.263 | 0.058 | 0.281 | 0.043 | 0.304 | 0.028 |
| NP (%) | 0.124 | 0.371 | -0.040 | 0.772 | 0.160 | 0.250 |
| Rapid (%) | 0.309 | 0.026 | 0.339 | 0.015 | 0.335 | 0.016 |
| Medium (%) | 0.278 | 0.045 | 0.280 | 0.044 | 0.313 | 0.024 |
| Slow (%) | -0.079 | 0.568 | -0.179 | 0.198 | -0.046 | 0.738 |
| **Kinematic parameters** (n=53) |  |  |  |  |  |  |
| VCL (µm/s) | 0.289 | 0.037 | 0.272 | 0.050 | 0.330 | 0.017 |
| VAP (µm/s) | 0.290 | 0.037 | 0.260 | 0.061 | 0.326 | 0.019 |
| VSL (µm/s) | 0.306 | 0.028 | 0.249 | 0.072 | 0.324 | 0.020 |
| STR (%) | 0.329 | 0.018 | 0.160 | 0.248 | 0.372 | 0.007 |
| LIN (%) | 0.323 | 0.020 | 0.206 | 0.137 | 0.379 | 0.006 |
| WOB (%) | 0.355 | 0.011 | 0.259 | 0.061 | 0.359 | 0.010 |
| ALH (µm/s) | 0.250 | 0.072 | 0.239 | 0.085 | 0.290 | 0.037 |
| BCF (Hz) | 0.274 | 0.048 | 0.235 | 0.090 | 0.352 | 0.011 |

**Supplementary Table 9**: Pearson’s correlations between colony demographics versus sperm morphometrics.

|  | **Brood pollen collected *versus*** | | **Amount of worker brood *versus*** | | **Total honey produced *versus*** | |
| --- | --- | --- | --- | --- | --- | --- |
|  | ***R*** | **P value** | ***R*** | **P value** | ***r*** | **P value** |
| Head length (µm) | 0.524 | 0.004 | 0.524 | 0.004 | -0.404 | 0.030 |
| Tail length (µm) | 0.136 | 0.481 | 0.136 | 0.481 | -0.618 | <0.001 |
| Total sperm length (µm) | 0.199 | 0.301 | 0.199 | 0.301 | -0.647 | <0.001 |
| Head:Tail ratio | 0.471 | 0.010 | 0.471 | 0.010 | -0.186 | 0.334 |

*r*, Pearson’s correlation coefficient
